# Citizen Science Provides Valuable Data for Monitoring Global Night Sky Luminance

**DOI:** 10.1038/srep01835

**Published:** 2013-05-16

**Authors:** Christopher C. M. Kyba, Janna M. Wagner, Helga U. Kuechly, Constance E. Walker, Christopher D. Elvidge, Fabio Falchi, Thomas Ruhtz, Jürgen Fischer, Franz Hölker

**Affiliations:** 1 Institute for Space Sciences, Freie Universität Berlin, Berlin, Germany; 2 Leibniz Institute for Freshwater Ecology and Inland Fisheries, Berlin, Germany; 3 National Optical Astronomy Observatory, Tucson, Arizona, USA; 4 National Oceanic and Atmospheric Administration, Boulder, Colorado, USA; 5 Light Pollution Science and Technology Institute (ISTIL), Thiene, Italy

## Abstract

The skyglow produced by artificial lights at night is one of the most dramatic anthropogenic modifications of Earth's biosphere. The GLOBE at Night citizen science project allows individual observers to quantify skyglow using star maps showing different levels of light pollution. We show that aggregated GLOBE at Night data depend strongly on artificial skyglow, and could be used to track lighting changes worldwide. Naked eye time series can be expected to be very stable, due to the slow pace of human eye evolution. The standard deviation of an individual GLOBE at Night observation is found to be 1.2 stellar magnitudes. Zenith skyglow estimates from the “First World Atlas of Artificial Night Sky Brightness” are tested using a subset of the GLOBE at Night data. Although we find the World Atlas overestimates sky brightness in the very center of large cities, its predictions for Milky Way visibility are accurate.

The development of personal computers, the global positioning system, mobile electronic devices, and above all the Internet, have enabled projects that would have seemed impossible two decades ago. A striking example of this was given by the successful identification of the locations of ten objects placed in the contiguous US in only 9 hours^1^. Citizen science projects are the scientific equivalent of crowdsourced projects like the Wikipedia and open street maps. The number and scope of such projects has increased greatly in recent years thanks to simplified geolocation and the Internet^2^,^3^. Some early projects involved the passive participation of interested citizens, who allowed their personal computers to be used as part of a distributed network to perform massive computations as part of the Search for Extraterrestrial Intelligence (SETI@home)^4^ or protein folding^5^. The success of these projects led to greater interaction between the participants and scientists, and citizen scientists have now classified the morphologies of hundreds of thousands of galaxies from the Sloan Digital Sky Survey^6^, predicted protein structures using the Foldit game^7^, and provided improved solutions to the Multiple Sequence Alignment problem of comparative genomics^8^. Teams of citizen scientists are now even designing new proteins, for example an enzyme with 18 fold increased activity^9^.

GLOBE at Night is a citizen science project related to light pollution, and has been running since 2006. The scientific goal of the GLOBE at Night project is to enable citizen scientists worldwide to quantify the degree of artificial skyglow at their location. Skyglow, a form of light pollution, is caused by the scattering of artificial light in the atmosphere. It is a major global environmental concern, both because of its known and potential ecological effects^10^,^11^,^12^,^13^, and because of the large amount of electrical energy required for its generation^14^. In stark contrast to the situation in daytime, the luminance of the night sky at locations on the Earth's surface is very poorly known, and the GLOBE at Night data aim to help patch this hole in our understanding of the biosphere. In addition to assembling a scientific data set, the GLOBE at Night project also aims to raise awareness of the economic costs and environmental impacts of skyglow among the citizen scientists who submit their observations.

Figure 1 demonstrates the difference in character between celestially lit (i.e. pristine) sites and artificially lit sites. The GLOBE at Night project makes use of this phenomenon to quantitatively classify the skyglow luminance by its relation to stellar visibility (“seeing”). However, many factors other than skyglow affect stellar visibility, for example the humidity and airmass in the direction of observation^15^. Some factors reduce stellar visibility by increasing the point spread function of stars (e.g. observer visual acuity^16^), some by reducing the signal to background ratio through the addition of direct (e.g. airglow) or scattered light (e.g. the Moon), and others through both mechanisms (e.g. aerosols). The GLOBE at Night project avoids the two most important factors by limiting the observations to times when the moon is set, and requiring observers to record the cloud cover. The rest of the variables, however, introduce a systematic uncertainty of unknown size into the dataset. In addition to physical phenomena, additional variance is introduced by the observational experience of observers^16^, as well as through mistakes made by participants during the data submission process, such as entering an innacurate location.

The goals of this paper are to estimate an upper bound on the systematic uncertainty associated with an individual naked eye GLOBE at Night sky luminance estimate, and to test the accuracy of the oft-cited “World Atlas of the artificial night sky brightness”^17^. This is achieved by comparing GLOBE at Night measurements to two approximate skyglow correlates, a satellite map of upward emitted light produced in 2010, and the World Atlas map of skyglow produced in 2001. By establishing the quantitative relationship between these maps and the GLOBE at Night data, we can test the deviation of individual GLOBE at Night observations from this prediction. Since it can reasonably be expected that deviations between the skyglow predictions and “true” skyglow introduce additional noise, the inherent uncertainty on any given GLOBE at Night measurement is assumed to be smaller than the observed deviation reported here.

## Results

### Principles

The Defense Meteorological Satellite Program Operational Linescan System observes light emitted upward from the Earth with a broadband sensor, with a spectral range extending into the infrared. The National Oceanographic and Atmospheric Administration (NOAA) has developed techniques to produce approximate maps of upward emitted radiance over most of the Earth's surface, and provided us with maps based on observations from 2010. This dataset is gridded in bins of 30″ by 30″, and is henceforth referred to as “DMSP”.

Light emitted upward into the atmosphere can be scattered by molecules or aerosols, producing skyglow. Emission data from a radiance calibrated DMSP map from 2001^18^ were used in conjunction with a radiative transfer model based on the work of Garstang^19^ to produce a “World Atlas of Artificial Night Sky Brightness”^17^, which provides worldwide estimates of skyglow luminance at zenith. Despite advances in satellite imaging and radiative transfer codes, the World Atlas dataset (henceforth WA) remains the state of the art in worldwide skyglow estimation. The World Atlas uses the same gridding as the DMSP, but due to uncertainties in the georeferencing of the 2001 data, the maps do not perfectly align. The WA maps of Europe and eastern and western North America were newly georeferenced to match the DMSP 2010 dataset.

The GLOBE at Night dataset consists of integer classifications of stellar visibility (naked eye limiting magnitude, henceforth NELM) from 1–7, and quantitative measurements taken using Sky Quality Meters (SQM)^20^. The data from 2009–2011 and 2012 were binned spatially to match the DMSP grid. Multiple observations were arithmetically averaged to produce a single GLOBE at Night data point per DMSP and WA pixel. The relationship between GLOBE at Night data and the DMSP and WA datasets was established using GLOBE at Night data from 2009–2011. Since a model cannot be properly evaluated using the data to which it was tuned, data from 2012 were kept “blind” until the entire analysis (including outlier removal) was finalized. Final assessment of the standard deviation of an individual observation is based on the March 2012 GLOBE at Night dataset.

### Observations

Profile histograms were produced by binning the GLOBE at Night data from 2009–2011 according to the logarithm of the DMSP and WA values. A linear function was fit to each of the histograms by minimizing the *χ*^2^ difference between the fit and each bin centroid, weighted by the standard deviation of the bin mean. (Higher order fits are inappropriate, since the analysis presumes that the map data represents true skyglow.) The results of the fits are shown in Table 1, with standard error of the fit parameter shown in parenthesis, and the quality of the fit shown in the *χ*^2^ per degree of freedom (*χ*^2^/dof). These fits can be used to estimate the faintest star that can be seen with the naked eye (NELM), or the sky radiance as measured by an SQM (in astronomical units of mag/arcsec^2^), for any given location. By evaluating the equations in Table 1 under conditions of negligible artificial skyglow, predictions for the dimmest visible stars or “natural” sky radiance as measured by the SQM are obtained.

**Figure 2:**
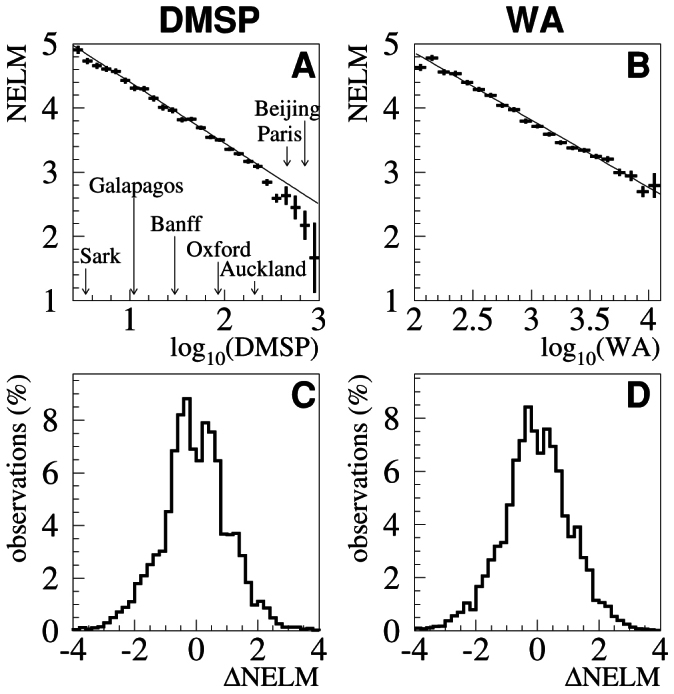
The relationship between average estimated naked eye limiting magnitude (NELM) and the satellite (DMSP, panel A) or skyglow atlas (WA, panel B) radiances are shown. Panels C and D show the fit residuals (i.e. histogram of differences between the reported GLOBE at Night values and those that would be predicted based on the fits in Table 1). Positive values in the fit residuals indicate skies that were darker than expected. The brightest DMSP values observed in several international locations are shown to provide context.

Figure 2 graphically demonstrates the relationship between the GLOBE at Night naked eye observations and the DMSP and WA maps. Panel A shows the average reported stellar visibility as a function of the logarithm of the upward directed light observed by the DMSP. Vertical error bars show the standard deviation of the bin mean, horizontal bars the bin width. To provide context, the value of the brightest DMSP pixel for several locations is shown. In decreasing brightness order these are the megacities Beijing and Paris, the city of Auckland in New Zealand, the town of Oxford, England, the village of Banff, Canada, the island San Christóbal in the Galapagos, and Sark Island, which is officially recognized as a “Dark Sky Island” by the International Dark Sky Association. It should be noted that the brightest areas occur in the city and town centers; adjacent and outlying locations in each city are necessarily darker than indicated. Lower values of naked eye limiting magnitude indicate that only bright stars can be seen.

Panel C of Figure 2 shows a histogram of the difference between the actual GLOBE at Night observations and the prediction for those locations according to the equations in Table 1, i.e.: 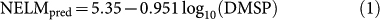
Negative residual values correspond to skyglow that was brighter than predicted. The non-zero width of this distribution is due to the systematic uncertainty of GLOBE at Night observation and the errors introduced by assuming that the DMSP values perfectly correlate with skyglow luminance. The right hand panels of Figure 2 are analogous to the left hand panels, only with the WA used as a skyglow correlate rather than the DMSP.

The standard deviation of the residuals (S*_R_*) between the observed GLOBE at Night naked eye observations and those predicted by the DMSP (similar to panel C) are shown for different subsets of the GLOBE at Night dataset in Table 2. The inherent uncertainty of a single GLOBE at Night observation based upon the March 2012 dataset was found to be S*_R_* = 1.2 stellar magnitudes.

**Figure 3:**
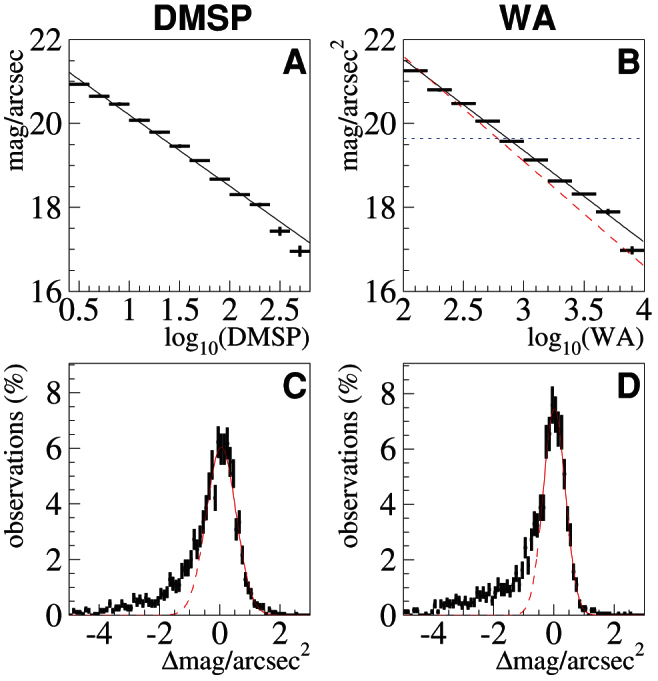
The relationship between SQM measured sky luminance and satellite observations (DMSP, panel A) and skyglow map (WA, panel B) is shown. Linear fits to the data are shown in black, and the dashed red line in panel B indicates the uncorrected estimate of the WA. The dotted blue line represents the approximate limit of Milky Way visibility. Panels C and D show the fit residuals (i.e. histogram of differences between the observed and predicted sky luminance). Positive values in the fit residuals indicate skies that were darker than predicted. Gaussian fits to part of the residual distributions are shown in C and D.

Figure 3 shows the relationship between the Sky Quality Meter measurements reported to GLOBE at Night from 2009–2012 and the location's brightness on the DMSP and WA maps. The panels are arranged as in figure 2, with the fit functions on top (panels A and C) and the fit residuals on the bottom (panels B and D). Since the World Atlas map estimates skyglow in approximately the same units measured by the SQM, it is possible to display the WA estimation in terms of mag/arcsec^2^. This is shown as the dashed red line in panel B. The approximate skyglow level at which the Milky Way is no longer visible to the naked eye is shown by the dotted horizontal blue line.

Tables 3 and 4 show the results of Gaussian fits to the residual peaks for the SQM data (c.f. Figure 3 for panels C and D respectively). Due to the large non-Gaussian tail on the negative side, the fits were performed over the range Δmag/arcsec^2^ > −0.5. The 2012 data is presented both with (2012 all) and without (2012 excl) a set of systematically shifted data, as described in the Methods.

## Discussion

The mean values of naked eye limiting magnitude reported by GLOBE at Night citizen scientists strongly correlates with both the observed values of emitted light measured by the DMSP worldwide, and with the estimates of the World Atlas of Artificial Night Sky Brightness for European and North American skyglow. This fact demonstrates once again that citizen scientists can indeed perform real scientific measurements and research^21^. While the uncertainty associated with any individual GLOBE at Night observation is very large (±1.2 stellar magnitudes), aggregated data provide a wealth of information. This is due to the law of large numbers; the mean of aggregated highly uncertain observations converges to the expectation value.

The large *χ*^2^/dof shown in Table 1 indicate that estimates for skyglow based on both the DMSP and World Atlas do not match “true” skyglow. This is not surprising, as the DMSP measures upward directed light (c.f. Supplemental Figure S8), the World Atlas is more than a decade old, and GLOBE at Night naked eye observations are not normally made at zenith. While statistically significant, the deviation from linear is very small compared to the variation in the data over the many magnitudes of skyglow brightness. Given this, all conclusions drawn here would be statistically indistinguishable from those that would be obtained using a higher order fitting function. The largest deviations from linear occur at both the bright and dim ends of the DMSP, as might have been expected since distant light sources affect skyglow but not the observed DMSP values.

The relationships between the datasets can be used to obtain predictions for what a typical observer would report in an area with no light pollution (c.f. Table 1). These predictions are very far from the established naked eye limiting magnitude for good observing sites, which is approximately 6 for an observer with low to average observing experience^16^. This is not surprising, as floor and ceiling effects in the GLOBE at Night methodology do not allow citizen scientists to underestimate brightness at artificially lit sites, or to overestimate stellar visibility at celestially lit sites. This is not a problem for data analyses, assuming that the functional relationship between aggregated GLOBE at Night observations and true skyglow can be discerned. The prediction for “natural” sky brightness based on the DMSP (21.9 mag/arcsec^2^) is quite a bit larger than that which is usually quoted, while the prediction based on the World Atlas (21.53 mag/arcsec^2^) is not far off of the 21.6 mag/arcsec^2^ assumed in the creation of the map.

In the cases where multiple GLOBE at Night observations were reported for a single location, the size of the standard deviation of the residuals was reduced (c.f. Table 2). Since S*_R_* reduced at a rate less than the square root of the number of observations, there appears to be an irreducible systematic uncertainty associated with either or both of the GLOBE at Night data and the method used here. One candidate explanation for this is the fact that the DMSP is a broadband sensor, which provides no information about the spectrum of local sources. Different lighting technologies produce radically different spectra^22^, and artificial sky radiance depends strongly on the wavelength under consideration^23^,^24^.

Even in the case of >30 observations, the uncertainty associated with the naked eye data from a single site means that observing a trend in the GLOBE at Night data at a single given location would take decades. The great value of GLOBE at Night as a program lies in its unique ability to monitor changes at a regional or global level through aggregation of data. Unfortunately, the locations for which GLOBE at Night data is available are not at all randomly distributed: far more data is available from the USA than any other region, and observation locations naturally strongly correlate with population centers. Additionally, changes in lighting practice (e.g. due to new technologies or legislation) are likely to have correlated effects over very large areas, and sites separated by only a few kilometers will share important skyglow sources. Any effort to monitor skyglow changes based on trends in GLOBE at Night time series will need to address these issues.

Some of the factors that affect seeing can be expected to result in year-to-year variations in the GLOBE at Night data at regional scales in light polluted areas (e.g. humidity and snow cover during the observation period). Since most of these factors are unlikely to vary in the same direction year-to-year for the whole Earth, the global trend in lighting should exhibit more stability, and should be easier to determine. This would be of scientific value, as the global rate of skyglow increase is highly uncertain^25^.

The World Atlas reported^17^ estimated skyglow brightness relative to an assumed “natural sky brightness” of 21.6 visual mag/arcsec^2^, or 252 *μ*cdm^−2^. The SQM spectral sensitivity does not perfectly match visual mag/arcsec^2^, but the agreement is close enough to evaluate the accuracy of the WA. The comparison was shown in Figure 3, and indicates that GLOBE at Night observations of night sky luminance are slightly darker than the decade old estimates of the World Atlas. For a city predicted to have a sky 10 times natural brightness, the skyglow observed is actually 8.8 times natural, and for the (highly uncommon) case of a megacity center estimated to have a sky 100 times brighter than natural, the skyglow is actually “only” 67 times brighter than natural. In evaluating these differences, it should be kept in mind that artificial skyglow is not stable, even over the course of a single night. In Berlin during the time period from 22:00 to 02:00, skyglow luminance is observed to decrease by approximately 40%^24^. Static measurements and estimates cannot adequately describe the actual light situation experienced over the course of a night, so future publications of skyglow estimates should follow the example of Aube and Kocifaj^26^, and report the time for which they are valid.

It seems unlikely that urban illumination has decreased since 2001. Indeed, well studied sites in northern Italy have experienced almost a doubling in installed flux since 1998. Despite this, the same sites have experienced very little change in skyglow due to light pollution laws that restrict direct uplight^27^. The angular distribution of urban uplight is a critical parameter for skyglow simulation, and the distribution used for the World Atlas may include too much horizontally directed light for some locations^28^. The uplight angular distribution remains the largest systematic uncertainty in skyglow simulations, and may be in a period of rapid change in response to laws designed to protect the natural night environment.

Other factors could also partially explain why the sky is not as bright as expected. For example, the DMSP dataset upon which the WA simulation is based is known to be biased bright at northern latitudes due to snow cover. Model simplifications which were necessary given the computing capabilities in 2001, particularly related to site elevation and atmospheric parameters, could also play a role. The World Atlas will be updated in the coming years based on an improved model^29^ and improved satellite data^30^ (due to an overpass time closer to midnight, the new data will not be as dependent upon winter data at high latitudes, when snow cover is common). The SQM data from GLOBE at Night will provide an important independent check of the improved model.

In addition to estimating sky brightness, the World Atlas also contained tables describing the fraction of the land area of each country where the Milky Way was no longer visible, and the fraction of the population for each country who could no longer view the Milky Way from where they live. The area over which the Milky Way is visible is important for conservation biology, as it was recently shown that nocturnal dung beetles, and presumably other insect species, use the Milky Way as for orientation^31^. Given the fairly good agreement between the WA estimation and the GLOBE at Night observations at the limit of Milky Way visibility (panel B of Figure 3), it is likely that the conclusions of Cinzano et al.^17^ for land area remain valid, at least in areas sampled by the GLOBE at Night observers.

Given that GLOBE at Night observations generally agree with location specific predictions based on satellite measurements, an obvious question is what the relevance of GLOBE at Night is, particularly in light of the capabilities of the new Suomi satellite^30^? The primary answer is that GLOBE at Night data measure something fundamentally different from satellite observations. Satellites observe upward directed light, and the amount of this light depends to a large extent on lighting policy. Changes in lighting technology, particularly a move to fully-shielded street lights, would be expected to impact downwelling skyglow and satellite observations of upwelling radiance very differently. The secondary answer is that satellite sensors are replaced on a timescale of a few years, whereas the evolution of the human eye proceeds much more slowly. Changes in satellite radiometric performance or spectral bands can pose challenges for time series analyses^32^. This problem is almost absent for a time series based on human vision, although an aging population will experience some changes in mean visual acuity and observational experience. If the GLOBE at Night dataset had a similar time span to the Christmas Bird Count of the Audubon Society^33^, we would now have a far better understanding of the history of changes in skyglow worldwide. Finally, like the DMSP, the nighttime sensor on Suomi doesn't match the human visual response. A multiband “Nightsat” mission would allow more accurate skyglow maps, and would provide a wealth of data for fields as disparate chronobiology and energy policy^34^, but the human eye will remain the ultimate arbitrator of stellar visibility.

As its time span increases, the GLOBE at Night dataset will become an increasingly useful tool in light at night research. Given the ubiquity of human eyes and smartphones, there is no fundamental reason why the GLOBE at Night project could not greatly expand in number of observations worldwide. The project offers students the chance to produce meaningful scientific data while learning about astronomy and light pollution, and would be a very worthwhile addition to science curricula.

In closing, we note that the ecological impact of light pollution is greatest on cloudy nights^35^. While the naked eye observations of GLOBE at Night are not possible on such nights, SQM measurements are. The results reported here suggest such measurements would be of great value in constraining and testing future cloudy night skyglow models, and demonstrate the large utility of collecting worldwide SQM measurements in a single database.

## Methods

### Datasets

Since 1992, NOAA has maintained a digital archive of data acquired by the Defense Meteorological Satellite Program Operational Linescan System. Observation of direct uplight of cities is an unintended, but extremely useful, byproduct of the instrument. Because the primary mission of the instrument is not scientific, radiance calibrated observations are not available with the exception of the years 2006 and 2010. A radiance calibrated dataset was also produced in 2001 for the production of the World Atlas of Artificial Night Sky Brightness^17^, but it is not publicly available.

The World Atlas converted the upward directed light measured by the DMSP into estimates of skyglow observed from the ground at zenith. In this analysis, we make use of the World Atlas data from 2001 for North America and Europe, and the 2010 DMSP-OLS radiance calibrated data. The World Atlas maps were newly georeferenced based on the 2010 DMSP-OLS data to ensure that differences between the two are due to differences in the data, and not due to difference in georeferencing. The World Atlas map contains estimates of artificial skyglow as a percentage of natural sky brightness. To account for celestial light we added 100 to each point in the WA map. The WA was chosen for comparison over direct estimates for naked eye limiting magnitude^36^ because of the greater conceptual simplicity of its linear scale (the relationship between WA estimates and true skyglow is ideally 1:1).

The GLOBE at Night project asks citizen scientists to go outside at least one hour after sunset and compare their view of a constellation to that shown in a series of star charts, which differ only in the maximum apparent magnitude (i.e. brightness) of the stars shown. The principle derives from earlier projects such as “How many Stars”^37^. In GLOBE at Night, the star charts are generated uniquely based on the observer's location, and examples for Berlin are shown as Supplemental Figures. By selecting the appropriate star chart, the participant's observation is converted into an estimate of the “naked eye limiting magnitude”, restricted to be an integer from 1–7 (c.f. Supplemental Figures S1–S7). This observation is then usually submitted via an online form. It has been shown that the dimmest star that observers report as visible depends strongly on observational experience^16^,^36^, and this should be expected to introduce dispersion into the GLOBE at Night data.

More quantitative observations can also be submitted to the GLOBE at Night project by citizen scientists who have access to a Sky Quality Meter (SQM)^20^, which is widely used for observations of skyglow^35^,^38^,^39^,^40^. The SQM measures approximate sky luminance in units of mag/arcsec^2^. This astronomical unit is commonly used in skyglow research, and can be thought of as a logarithmic measure of sky *darkness*. An increase of 5 in mag/arcsec^2^ corresponds to a sky that is 100 times darker. It is possible to *approximately* convert mag/arcsec^2^ into SI units of cd/m^2^ using the formula cd/m^2^ = 9.0 × 10^4^ × 10^−0.4*x*^, where *x* is the luminance in mag/arcsec^2^ (equation from Paul Schlyter: www.stjarnhimlen.se/comp/radfaq.html). To avoid introducing unnecessary conversion errors, and because we perform analysis on a logarithmic scale, results are reported using mag/arcsec^2^ as directly measured by the SQM.

The observation directions for each of the different datasets is shown in Supplemental Figure S8. The only perfect correspondence between the datasets is between the estimates of the skyglow World Atlas (which are for zenith) and the SQM observations of GLOBE at Night. The visual observations of GLOBE at Night are not usually taken at zenith, and the DMSP satellite observes upward rather than downward directed light.

### Analysis

Data from the GLOBE at Night campaigns was filtered to remove any observations where citizen scientists reported clouds, as well as reports where the word “snow” was entered into the free form text box. Data from 2011 for which the constellation was not reported were also removed.

The individual data points from the GLOBE at Night campaigns of 2009–2011 were divided into spatial bins defined by the DMSP map pixels. In cases when more than one observation existed in a bin, the number of observations and their mean were recorded. These data were analyzed with ArcGIS 9.3.1 to produce a table containing the DMSP radiance, WA radiance, and GLOBE at Night observation in each unique location. These data were then binned in profile histograms according to the logarithm of the DMSP or WA data, and the vertical error bars were set to the standard deviation of the mean. The resulting histograms were fit by minimizing the *χ*^2^ differences between a linear function and the bin centroids, weighted according to the bin's standard error. Observations from areas where the DMSP map reported zero light could not be used, as the logarithm was not defined.

The SQM data was found to include a long non-Gaussian tail, which presumably represents observations in which the citizen scientist was too close to an artificial light source (the tail extends only in the direction of brighter than expected skies). To prevent this data from artificially reducing the mean value (and having the situation where the peak observation is not centered with the mean), we first identified all SQM observations with a residual greater than 1.5*σ* away from the peak. We then produced new profile histograms with these data rejected, and these were fit with a linear function to obtain the equations listed in Table 1. The effect of this was to center the residuals close to zero, with only a minor effect on the fit slope. Because the fit residuals had a non-Gaussian tail on one side, we determined their width by performing Gaussian fits over the range Δmag/arcsec^2^ > −0.5.

In order to have an independent dataset for evaluating the uncertainty inherent in a single observation, this procedure and the resulting fit functions were finalized before the 2012 GLOBE at Night data was examined. Since the GLOBE at Night campaign in 2012 included additional months of the year, it was decided in advance that the standard deviation of the fit residuals from March 2012 would be reported as our final result. The uncertainty is defined based on the DMSP dataset rather than the WA because it covered a larger spatial extent. The analysis assumes that the global change in sky brightness over a four year period is small compared to the standard deviation of the residuals. This assumption is justified based on the estimated worldwide trends in lighting^25^.

After “opening the box” and examining the data from 2012, we found a set of SQM data which differed systematically from the rest of the GLOBE at Night data. This set of data reported values 0.4 mag/arcsec^2^ larger (darker) than average, and interestingly with a much smaller than average standard deviation, *σ* = 0.13. This entire subset of the data was taken by two of the authors (Kyba and Ruhtz) on a single night from an SQM mounted on top of a vehicle traveling on a German Autobahn. We can think of two possible sources of the discrepancy. First, in order to avoid contamination of lights near the Autobahn and changes in orientation of the SQM, we selected the darkest observation from each 15 second interval. The SQM is known to be strongly affected by external electromagnetic fields, and their presence combined with our procedure would necessarily introduce a dark shift. Second, the data were taken on an unusually clear night after midnight. These two environmental factors both act to darken the artificially lit night sky. Since the data were taken in a systematically different way from all other GLOBE at Night data, we felt justified in removing them from our analysis. In keeping with the standards required for performing a blind analysis, we present both our final results and the results we found upon immediate inspection of the blind data.

## Author Contributions

C.C.M.K., J.F. and F.H. conceived the study. C.C.M.K., J.M.W. and H.U.K. analyzed the data. C.E.W. organized GLOBE at Night, and C.C.M.K., J.M.W., H.U.K., C.E.W. and T.R. contributed data to GLOBE at Night. C.D.E. provided DMSP data, and C.D.E. and F.F. provided the World Atlas data. All authors discussed the results and commented on the manuscript.

## Supplementary Material

Supplementary Information Supplementary figures

## Figures and Tables

**Figure 1:**
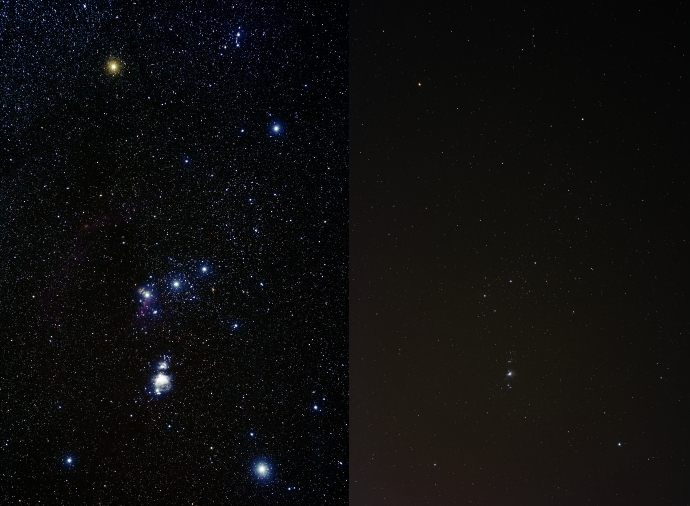
Skyglow reduces the visibility of celestial objects for both the human eye and consumer cameras. The GLOBE at Night project works by assessing the visibility of stars near constellations like Orion, shown here. This image, entitled “Light pollution: it's not pretty” was produced by Jeremy Stanley and is released under the CC BY 2.0 license (http://commons.wikimedia.org/wiki/File:Light_pollution_It%27s_not_pretty.jpg), access date 18 January 2013.

**Table 1:** These equations describe the best fit linear relationship between naked eye observations (NELM) and the map values of the DMSP and WA (top), and between SQM measurements and the maps (bottom). The estimated uncertainty in the fits are shown in parenthesis. The fits can be used to predict the naked eye star visibility or SQM measured sky radiance in the case of no artificial skyglow (0LP prediction). For the DMSP, the y-intercept represents almost no observed light, while for the WA zero artificial skyglow occurs at x = 2 (the value 25.89 has no physical significance). The large *χ*^2^ per degree of freedom in the fits indicates that the fit does not perfectly match the data

Data	Skyglow Correlate	Intercept	Slope	0LP prediction	*χ*^2^/dof
NELM	2010 DMSP	5.35 (0.03)	−0.951 (0.016)	5.35 (0.03) Vmag	52.7/24
NELM	World Atlas	6.96 (0.06)	−1.05 (0.02)	4.86 (0.02) Vmag	32.4/19
SQM	2010 DMSP	21.90 (0.04)	−1.69 (0.02)	21.90 (0.04) (mag/arcsec^2^)	37.7/10
SQM	World Atlas	25.89 (0.07)	−2.18 (0.03)	21.53 (0.03) (mag/arcsec^2^)	19.9/8

**Table 2:** The difference between the GLOBE at Night observations and the values predicted by the fit to the DMSP data are shown for different selections of the GLOBE at Night data. In each case, the number of unique locations sampled, mean value, and standard deviation of the data from the prediction (S*_R_*) are shown. The data are alternatively broken down by the two week period in which they were acquired (top), or by the total number of observations (obs) at a single location (bottom)

Dataset	Unique locations	Mean	S*_R_*
2009–2011	13891	0.00	1.06
2009 Mar	3530	0.07	1.11
2010 Mar	3600	0.14	1.13
2011 Feb–Mar	2827	−0.17	0.96
2011 Mar–Apr	1146	−0.41	1.27
2012 Jan	794	−0.09	1.16
2012 Feb	706	−0.15	1.10
2012 Mar	1057	0.00	1.16
2012 Apr	746	−0.28	1.38
1 obs	13621	−0.03	1.13
2 obs	2390	0.02	0.99
3–6 obs	1825	−0.06	0.94
7–12 obs	417	−0.15	0.85
13–20 obs	110	−0.07	0.57
21–30 obs	41	−0.06	0.54
>30 obs	50	−0.06	0.45

**Table 3:** Results of fitting a Gaussian function to the differences between observed SQM values and those expected based on the DMSP radiance observed at that location. The 2009–2012 dataset excludes a portion of the 2012 data, as described in the Methods

Dataset	Observations	Mean	Fit *σ*	*χ*^2^/dof
2009–2011	1243	0.00	0.51	21.6/24
2012 all	863	0.27	0.49	30.0/21
2012 excl	705	0.11	0.45	28.6/21
2009–2012	1948	0.05	0.49	33.0/25

**Table 4:** Results of fitting a Gaussian function to the differences between observed SQM values and those expected based on the sky luminance expected using the World Atlas for that location. The “2012 all” dataset was not normally distributed, as is indicated by the poor *χ*^2^/dof, and the standard error in this case is larger than *σ*

Dataset	Observations	Mean	Fit *σ*	*χ*^2^/dof
2009–2011	1130	0.01	0.35	23.4/19
2012 all*	771	0.13*	0.33*	67.3/16
2012 excl	605	0.02	0.35	19.3/16
2009–2012	1735	0.01	0.37	21.0/23
