# Supplementary material for: Citizen Science Provides Valuable Data for Monitoring Global Night Sky Luminance
Source: Sci Rep. 2013 May 16;3:1835. doi: 10.1038/srep01835 (PMC3655480; doi:10.1038/srep01835)
Supplement: Supplementary Information — Supplementary figures [file srep01835-s1.pdf]

Citizen Science Provides Valuable Data for Monitoring Global Night Sky Luminance

Christopher C. M. Kyba, Janna M. Wagner, Helga U. Kuechly, Constance E. Walker, Christopher D. Elvidge, Fabio Falchi, Thomas Ruhtz, Jürgen Fischer, Franz Hölker

Supplementary Information

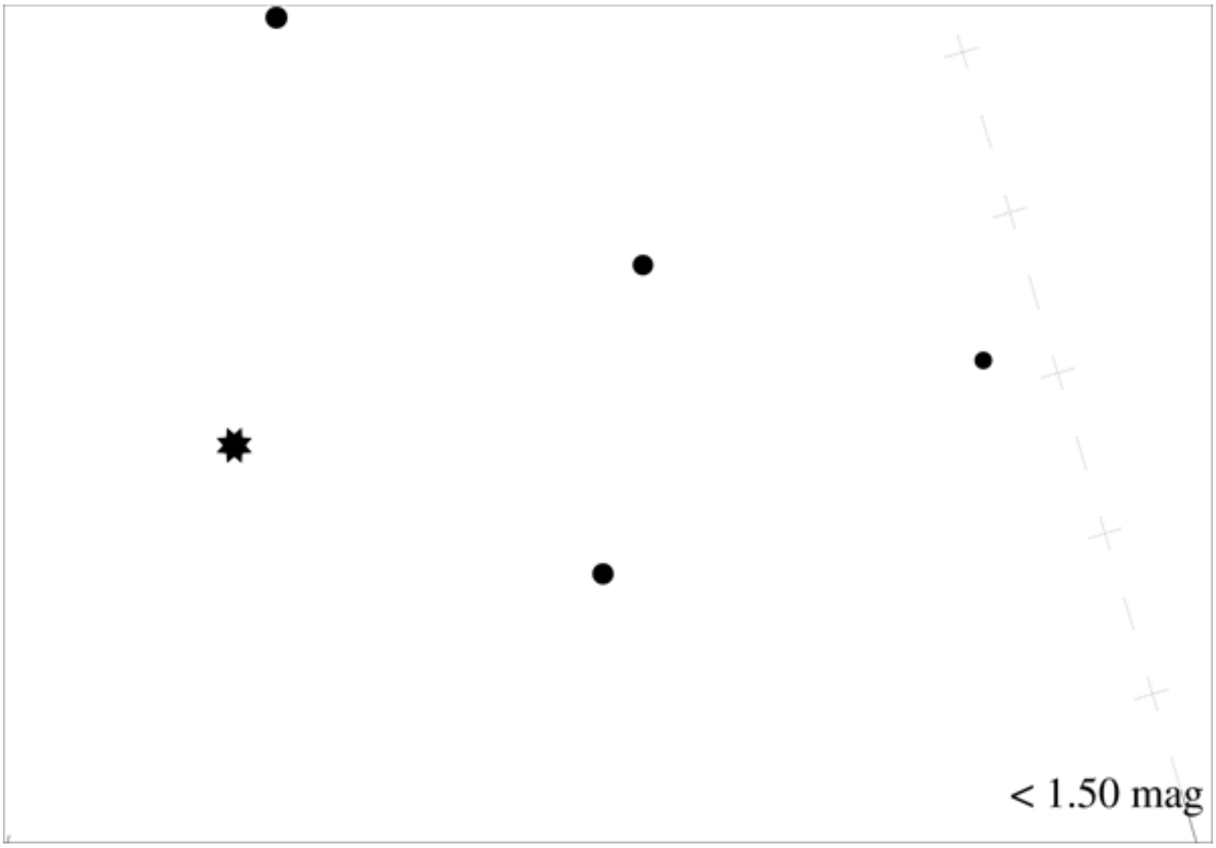

Supplemental Figure S1: GLOBE at Night star chart for limiting magnitude 1.

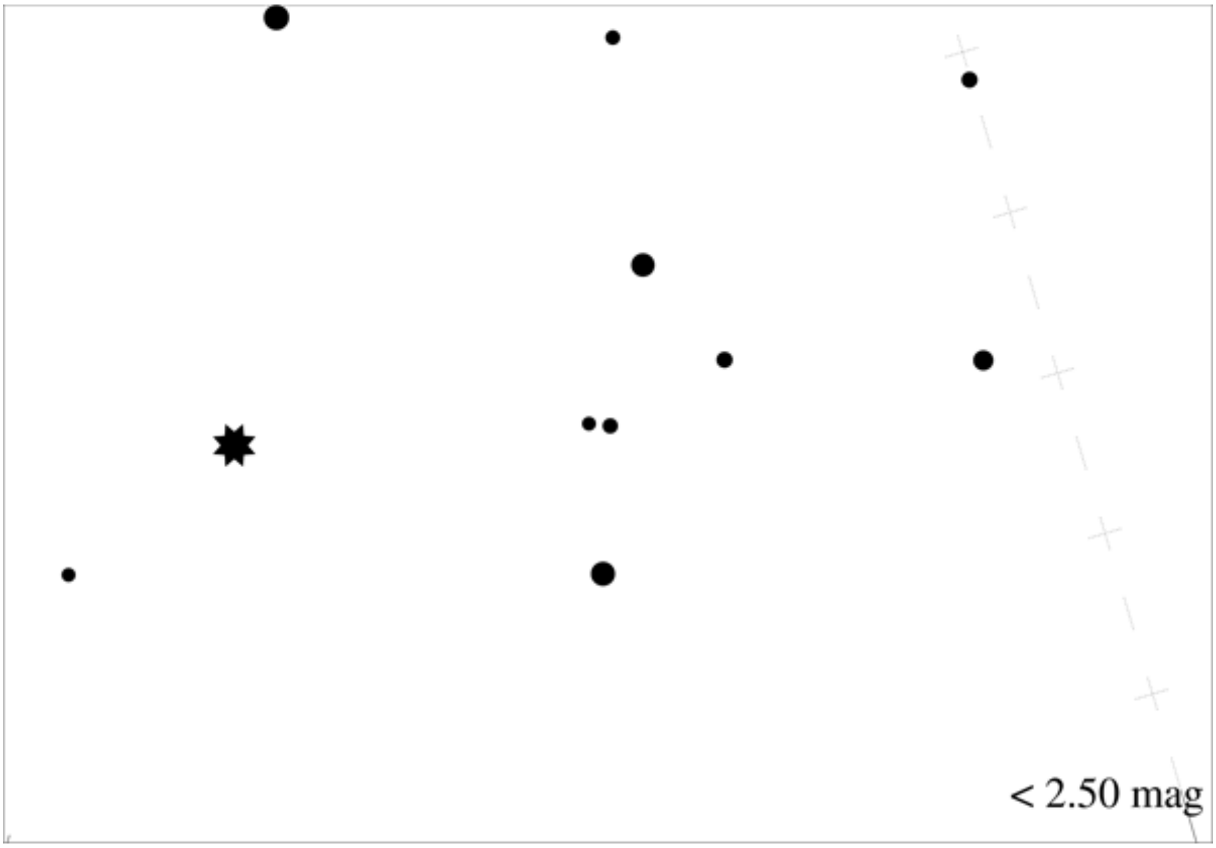

Supplemental Figure S2: GLOBE at Night star chart for limiting magnitude 2.

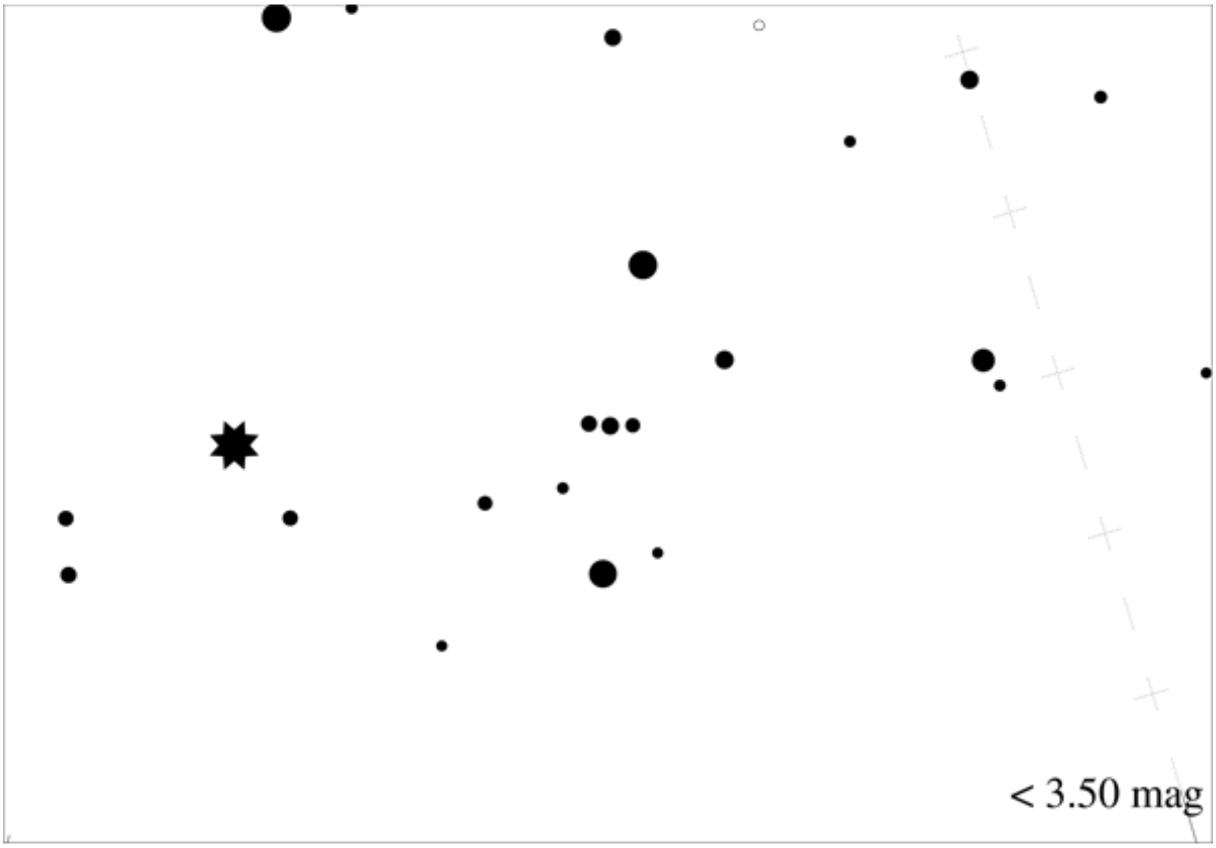

Supplemental Figure S3: GLOBE at Night star chart for limiting magnitude 3.

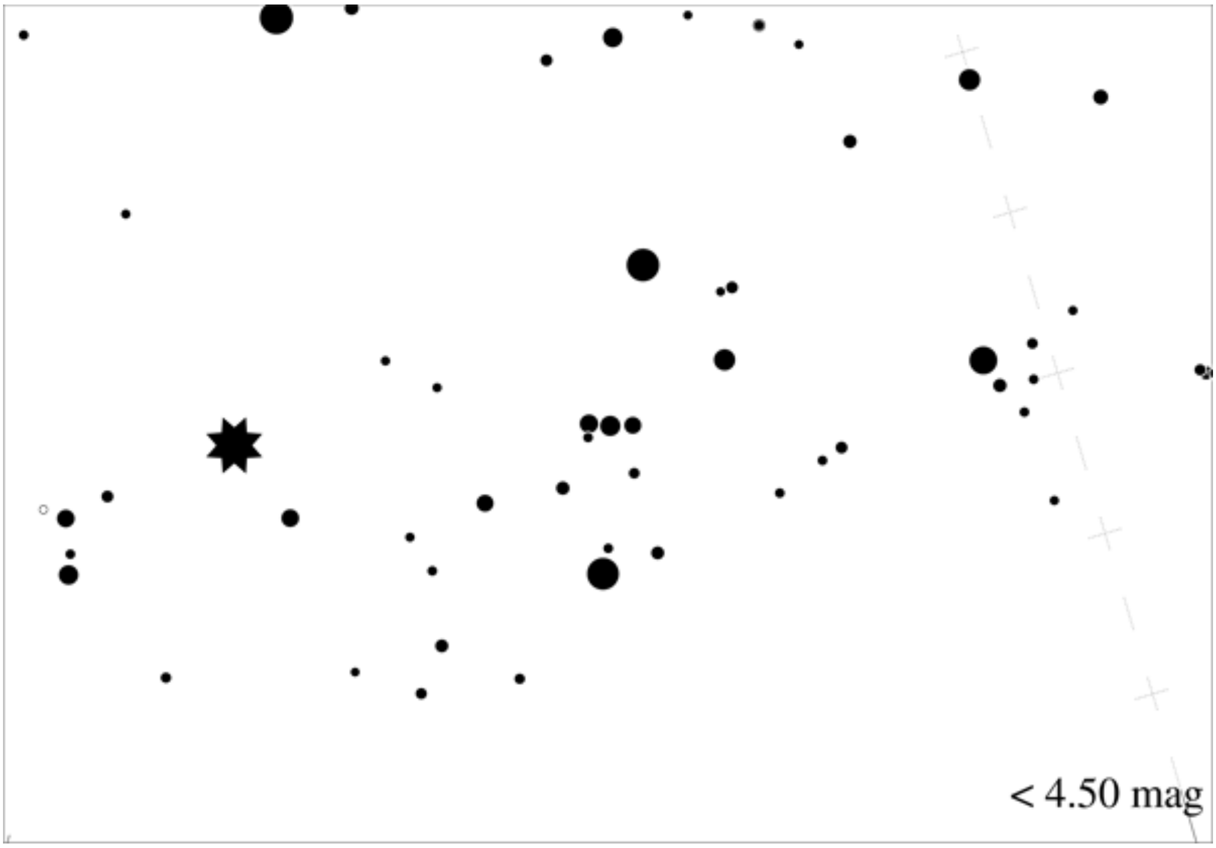

Supplemental Figure S4: GLOBE at Night star chart for limiting magnitude 4.

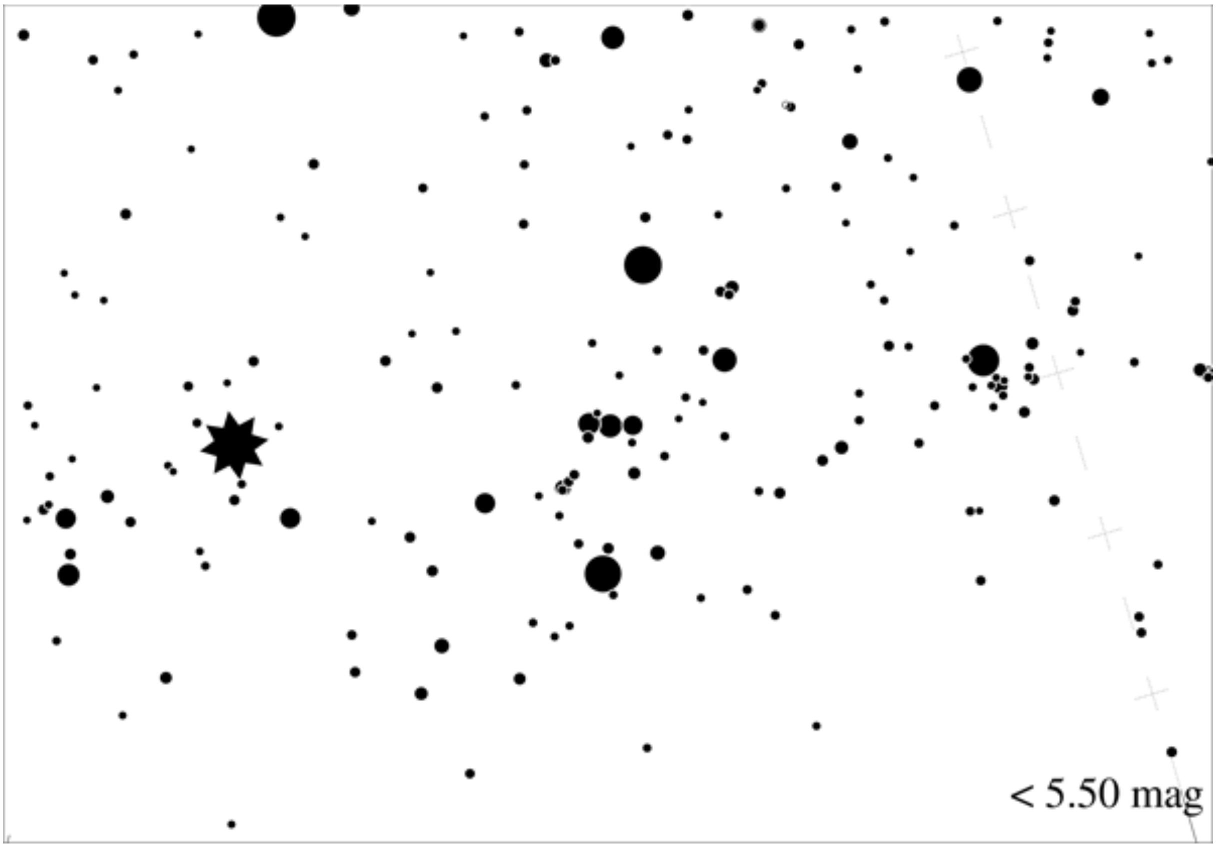

Supplemental Figure S5: GLOBE at Night star chart for limiting magnitude 5.

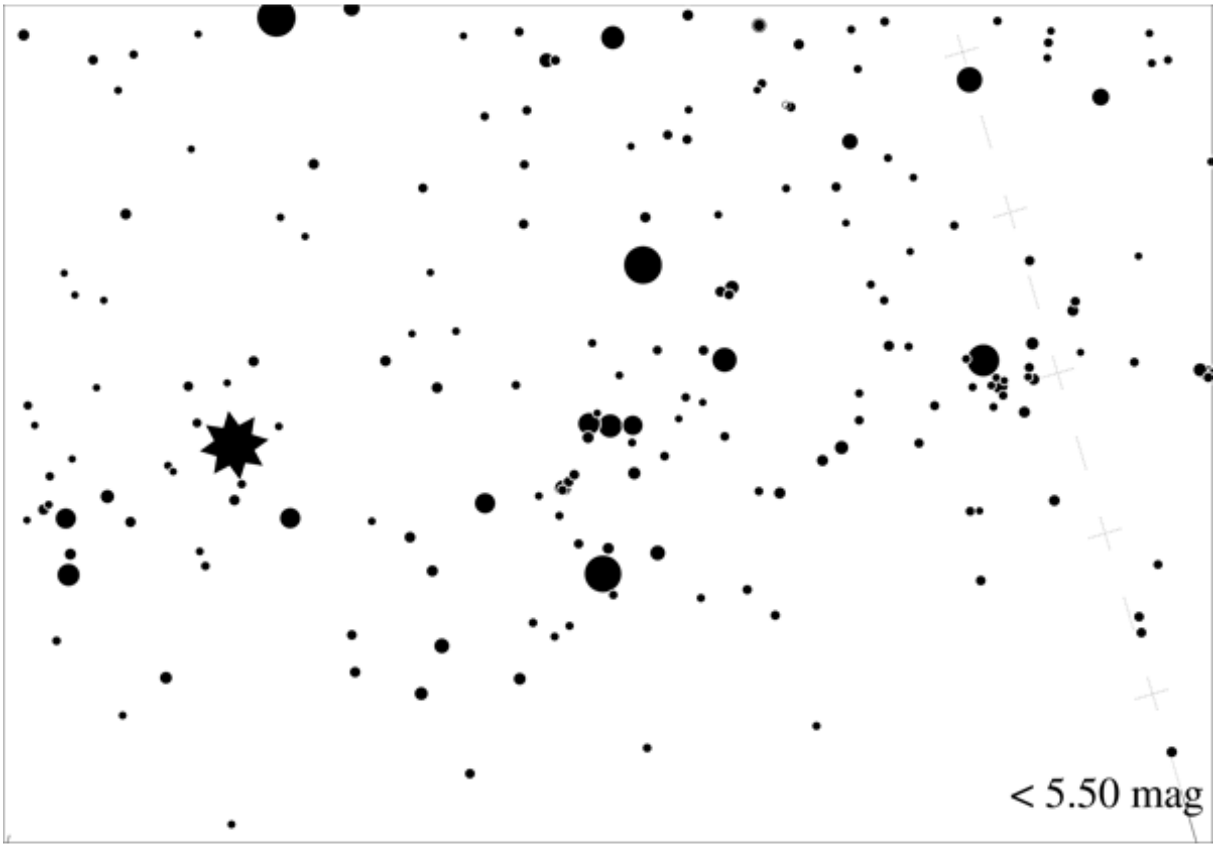

Supplemental Figure S6: GLOBE at Night star chart for limiting magnitude 6.

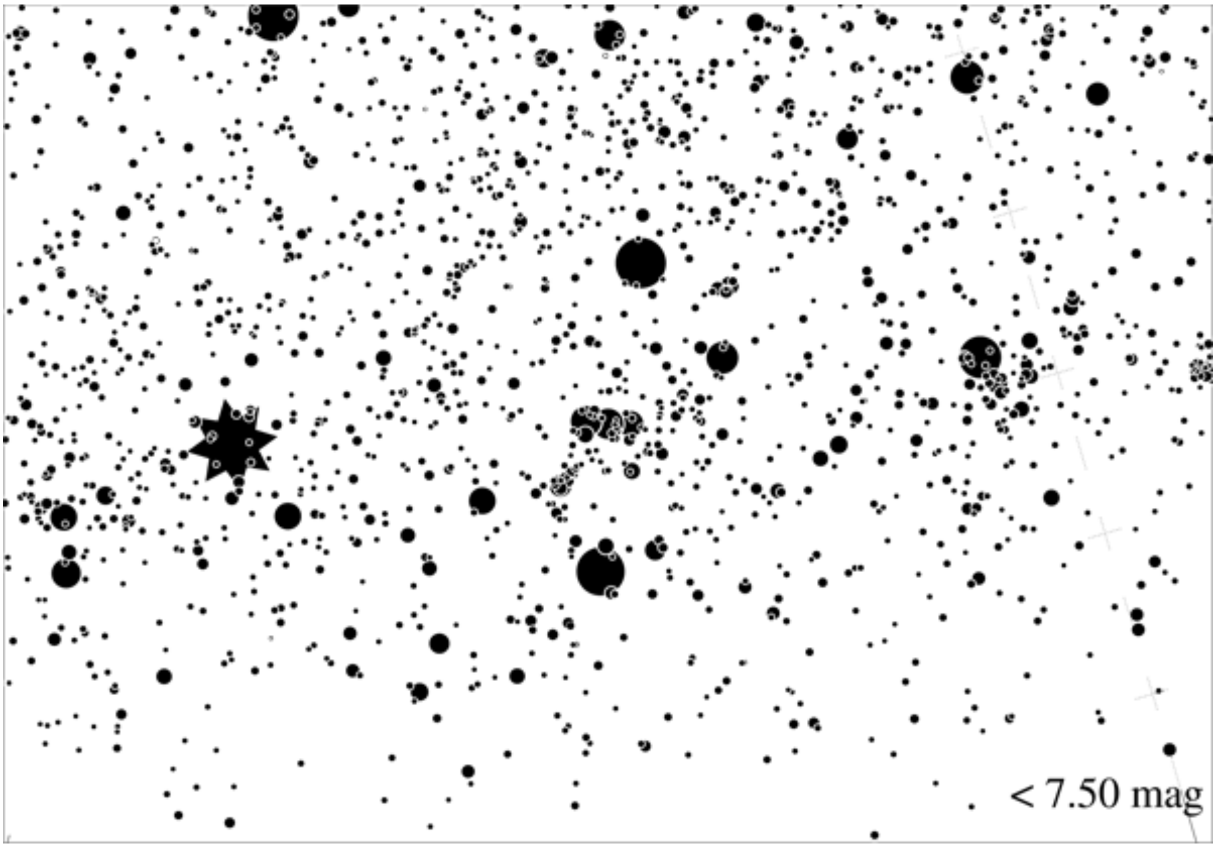

Supplemental Figure S7: GLOBE at Night star chart for limiting magnitude 7.

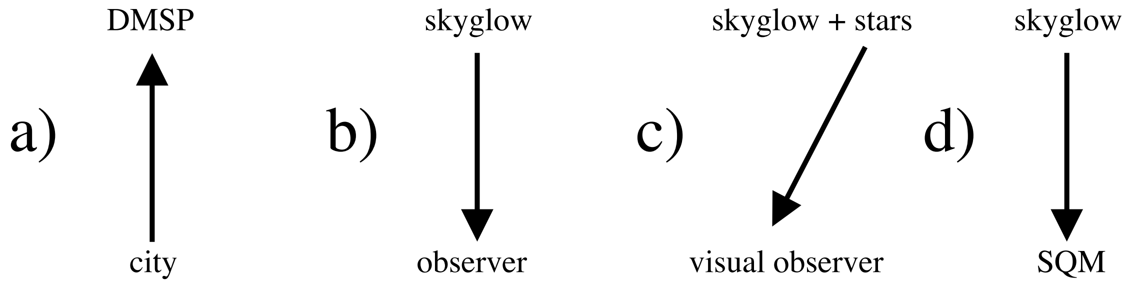

Supplemental Figure S8: The observing directions for the DMSP satellite map (a), World Atlas skyglow map (b), GLOBE at Night visual observations (c), and GLOBE at Night SQM observations (d).
